# Gross on Foreign Bodies in the Air-Passages

**Published:** 1854-12

**Authors:** 


### GROSS ON FOREIGN BODIES IN THE AIR-PASSAGES.

A copy of this work has been received, but it came to hand too late to receive more than a brief notice in our present number. We are able, however, after a very cursory examination, to say that it is characterized by the spirit of research for which its learned author is so distinguished. It presents a summary of our professional experience on the subject of foreign bodies in the air-passages, and the student who wishes to learn what has been suffered or done in such cases will find it the shortest course to refer to this work of Prof. Gross. As a work of reference it possesses the highest value, for in addition to the facts gleaned from all our extant medical literature, it embodies the practical experience of the author himself.

